# Zoonotic Infection with Oz Virus, a Novel Thogotovirus

**DOI:** 10.3201/eid2802.211270

**Published:** 2022-02

**Authors:** Ngo T.B. Tran, Hiroshi Shimoda, Keita Ishijima, Kenzo Yonemitsu, Shohei Minami, Supriyono, Yudai Kuroda, Kango Tatemoto, Milagros V. Mendoza, Ryusei Kuwata, Ai Takano, Masahiko Muto, Kyoko Sawabe, Haruhiko Isawa, Daisuke Hayasaka, Ken Maeda

**Affiliations:** Yamaguchi University, Yoshida, Japan (N.T.B. Tran, H. Shimoda, S. Minami, Supriyono, A. Takano, D. Hayasaka, K. Maeda);; National Institute of Infectious Diseases, Toyama, Tokyo, Japan (K. Ishijima, K. Yonemitsu, Y. Kuroda, K. Tatemoto, M.V. Mendoza, K. Sawabe, H. Isawa, K. Maeda);; Okayama University of Science, Imabari, Japan (R. Kuwata);; Yamaguchi Prefectural Grand Medical Center, Hofu, Japan (M. Muto)

**Keywords:** Oz virus, zoonoses, thogotoviruses, tick-borne viruses, vector-borne infections, arboviruses, viruses, Japan

## Abstract

Oz virus is a novel thogotovirus isolated from ticks that causes lethal infection in mice. We conducted serosurveillance of Oz virus infection among humans and wild mammals in Japan using virus-neutralization tests and ELISAs. Results showed that Oz virus may be naturally infecting humans and other mammalian hosts.

The genus *Thogotovirus*, family *Orthomyxoviridae,* comprises viruses that are most frequently transmitted by a variety of hard and soft tick species (*1*). Although most thogotoviruses are associated with tick species, there are several exceptions, such as Sinu virus, which was isolated from mosquitoes (*2*); Dielmo orthomyxovirus, isolated from *Culicoides* midges (*3*); and Araguari virus, isolated only from vertebrates (*4*). Thogoto, Dhori, and Bourbon viruses have been associated with human disease. Thogoto and Dhori viruses have been reported to cause encephalitis, febrile illness, and death in humans (*5*,*6*), and Bourbon virus to cause febrile illness and death in humans (*7*). In addition, Thogoto virus has been reported to cause abortions in sheep (*8*), and many wild animals are positive for Bourbon virus antibodies (*9*).

Oz virus, a new member of the genus *Thogotovirus*, was first isolated from a pool of 3 *Amblyomma testudinarium* tick nymphs collected in Ehime prefecture, Japan (*10*). Phylogenetic analyses revealed that Oz virus is more closely related to Dhori, Batken, and Bourbon viruses than to other thogotoviruses (*10*). In addition, Oz virus has been shown to cause lethal infection in experimentally challenged suckling mice. To determine the potential of Oz virus as a zoonotic pathogen, we performed serosurveillance of Oz virus infection among mammals, including humans, in Japan.

## The Study

To examine whether mammals are naturally infected with Oz virus, we collected serum samples from 24 hunters and 240 wild animals (40 Japanese macaques [*Macaca fuscata*], 124 wild boars [*Sus scrofa*], and 76 sika deer [*Cervus nippon*]) captured in Yamaguchi prefecture, Japan, during 2013–2019. Because Yamaguchi prefecture is close to Ehime prefecture and the environment in Yamaguchi is very similar to that in Ehime, we used stocked samples in Yamaguchi prefecture for the first surveillance of Oz virus infection. To test for the presence of Oz virus antibodies in the serum samples, we performed a PRNT_80_ (80% plaque-reduction neutralizing test) using Oz virus (Table 1). Among humans, 8.3% of the serum samples had Oz virus neutralization (VN) antibodies; VN titers were 1:40 and 1:80. In wild animals, serum from 47.5% of macaques, 60.5% of wild boars, and 73.7% of sika deer in Yamaguchi had Oz virus VN antibodies (Table 1).

**Table 1:** Serosurveillance of Oz virus infection by virus-neutralization test among mammals in Yamaguchi prefecture, Japan

Species	Genus and species	Years	Virus-neutralization titer						
			<1:10	1:10	1:20	1:40	1:80	1:160	>1:160
Human	*Homo sapiens*	2015	22	0	0	1	1	0	0
Macaque	*Macaca fuscata*	2018–2019	21	0	2	3	3	6	5
Wild boar	*Sus scrofa leucomystax*	2013–2014	49	2	12	10	15	20	16
Sika deer	*Cervus nippon*	2014–2015	20	5	8	11	12	13	7

We applied ELISA protocol used for serosurveillance of many infectious diseases (*11*–*13*) to detect Oz virus antibodies in the serum samples from wild animals. We extracted proteins from Oz virus or mock-infected Vero cells and used the extracts as ELISA antigens. To prepare the primary antibody, we diluted serum samples 1:100 in phosphate-buffered saline containing 0.05% Tween 20 and 0.4% Block Ace. We used peroxidase conjugated recombinant protein A/G (Thermo Fisher Scientific, https://www.thermofisher.com) as the secondary antibody and KPL ABTS peroxidase substrate (SeraCare Life Sciences, https://www.seracare.com) as the detection reagent. We measured absorbance using a spectrophotometer (Bio-Rad Laboratories, https://www.bio-rad.com) with a 405 nm filter and subtracted the value of the corresponding control mock-infected cells from all values.

**Figure 1:**
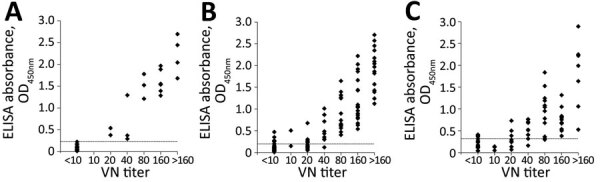
Dot plot comparison between VN test and ELISA against Oz virus in serum samples from wild animals in Yamguchi prefecture, Japan. A) Macaques (n = 40); B) wild boar (n = 124); C) sika deer (n = 76). The correlation coefficient between VN test and ELISA from macaques was 0.9163, from wild boars was 0.8807, and from sika deer was 0.7569. The optimal cutoff value of ELISA was calculated by 2-graph receiver-operating characteristic curve. The optimal cutoff values were set at 0.225 for macaques, 0.197 for wild boar, and 0.317 for sika deer serum samples and are indicated by dotted lines.

To determine ELISA cutoff values, we tested serum samples from the 40 macaques, 124 wild boars, and 76 sika deer captured in Yamaguchi prefecture. We compared the optical density values of the ELISA to the results of the VN test by 2-graph receiver-operating characteristic (ROC) curve analysis as described elsewhere (*14*). In macaques, the correlation coefficient between the ELISA and VN test was 0.9163, and an ELISA cutoff value of 0.2245 produced 100% sensitivity and specificity. In wild boars, the correlation coefficient was 0.8807, with 88.0% sensitivity and 89.8% specificity at an ELISA cutoff value of 0.1965. In sika deer, the correlation coefficient was 0.7569, sensitivity 78.6%, and specificity 80.0% at an ELISA cutoff value of 0.3165 (Figure 1).

**Figure 2:**
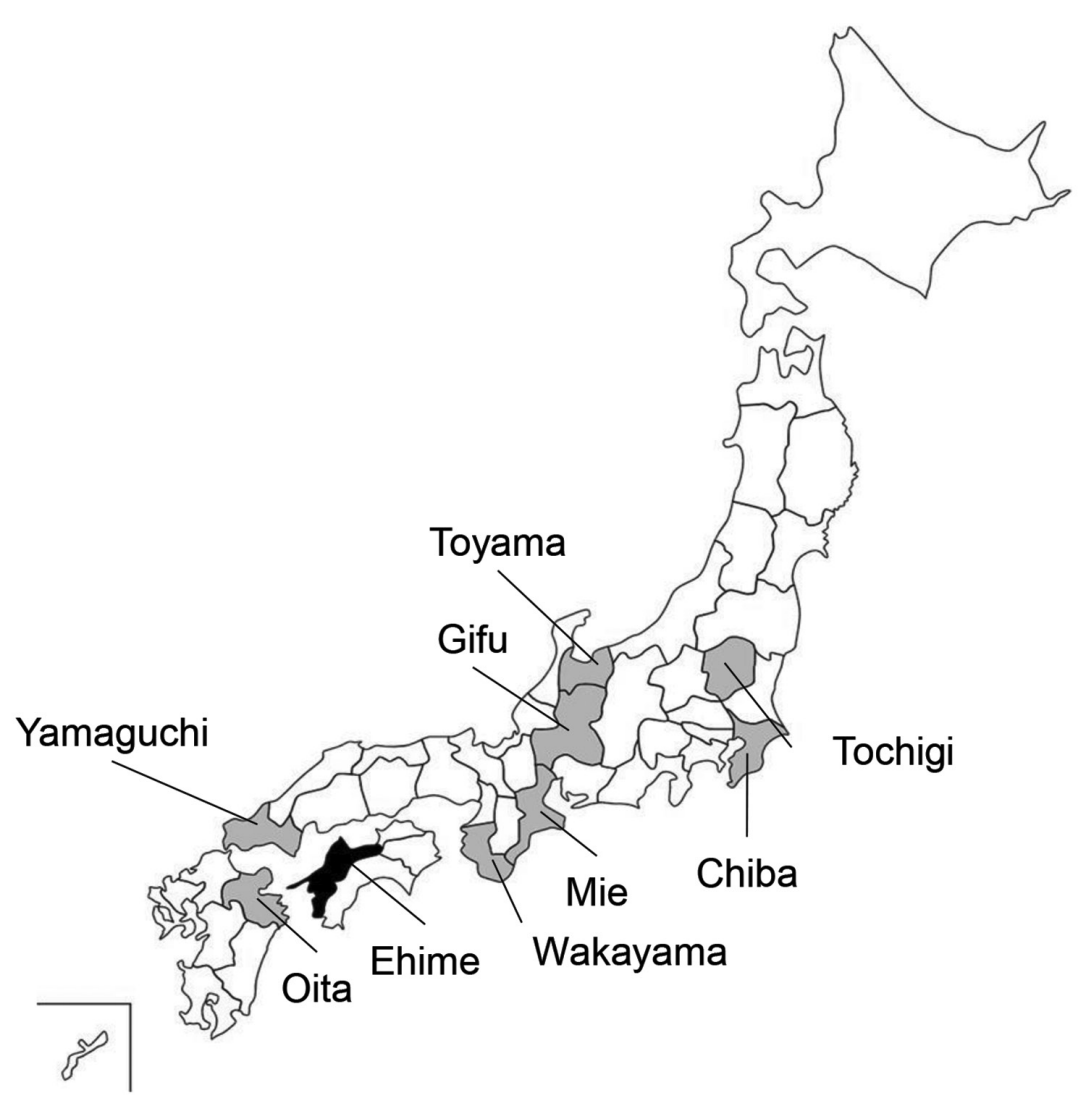
Collection sites of serum samples from macaques, wild boars, and sika deer for study of Oz virus seroprevalence in Japan. Gray shading indicates prefectures in which samples were collected; black shading indicates Ehime prefecture, where Oz virus was first isolated.

Next, we surveyed Oz virus infection among macaques, wild boars, and sika deer in many prefectures in Japan using the established ELISA (Table 2; Figure 2). Among 197 macaques captured during 2007–2019, seropositivity rates were 47.5% in Yamaguchi, 33.3% in Wakayama, and 6.3% in Mie prefectures. Among 879 wild boars captured during 2007–2014, seropositivity rates were 10.3% in Oita, 55.8% in Yamaguchi, 34.8% in Wakayama, 10.5% in Gifu, and 0% in both Toyama and Tochigi prefectures. Among 450 sika deer, seropositivity rates were 37.8% in Yamaguchi, 11.1% in Wakayama, 8.3% in Gifu, and 30% in Chiba prefectures.

**Table 2:** Serosurveillance of Oz virus infection by ELISA among wild animals, Japan

Species	Prefecture	Years	Cutoff	No. serum samples examined	No. (%) positive serum samples
Macaque	Yamaguchi	2018–2019	0.225	40	19 (47.5)
	Wakayama	2012–2013		15	5 (33.3)
	Mie	2007		142	9 (6.3)
Wild boar	Oita	2012	0.197	58	6 (10.3)
	Yamaguchi	2010–2014		344	192 (55.8)
	Wakayama	2007–2013		89	31 (34.8)
	Gifu	2014		19	2 (10.5)
	Toyama	2014		20	0
	Tochigi	2010–2012		349	0
Sika deer	Yamaguchi	2010–2015	0.317	407	154 (37.8)
	Wakayama	2010–2014		9	1 (11.1)
	Gifu	2014		24	2 (8.3)
	Chiba	2014		10	3 (30.0)

First, we applied PRNT_80_ to detect Oz virus antibodies in humans and wild animals in Yamaguchi prefecture. The results showed that 60.5% of wild boars, a major host of *A. testudinarium*, and 73.7% of sika deer in Yamaguchi prefecture during 2013 and 2015 had Oz virus VN antibodies, indicating that the virus was infecting wild animals in the western part of Japan. Next, we examined wild macaques for Oz virus infection; 48% were infected, and the antibody titers were high. In addition, 2 persons who hunted wild boars and sika deer in Yamaguchi prefecture had Oz virus antibodies. These results indicate that humans and macaques are also exposed to Oz virus. 

We compared results from an ELISA, established for this study, using an Oz virus–infected cell extract for the surveillance of Oz virus infection among many mammalians, with those from the VN test to determine correlation between the 2 tests. The correlation coefficient was 0.9163 for macaques, 0.8807 for wild boars, and 0.7569 for sika deer, suggesting that the ELISA is effective for serosurveillance of Oz virus infection in samples from many animal species. However, because its sensitivity and specificity differed among animal species and values were lower for sika deer, in particular, cutoff values should be determined for each animal species. In addition, VN testing should be performed to confirm the presence of Oz virus antibodies.

Our nationwide surveillance of Oz virus infection in Japan indicated that many wild animals were positive for Oz virus antibodies. However, wild boars in Toyama and Tochigi prefectures did not have Oz virus antibodies, suggesting that the virus might not be distributed in the northern and eastern parts of Japan. *A. testudinarium* is the major tick species that infests humans in the southern and western parts of Japan (*15*), and because we found Oz virus mainly in those areas, it appears that the distribution of Oz virus–infected animals correlates with the habitat of the tick. In addition, because 2 hunters in Yamaguchi prefecture tested positive for Oz virus antibodies, further investigation is needed to determine whether Oz virus might be a zoonotic pathogen, especially because intracerebral inoculation of the virus in suckling mice causes lethal disease (*10*). 
